# Endoscopic removal of an unexpected colon foreign body

**DOI:** 10.1055/a-2500-3050

**Published:** 2025-01-14

**Authors:** Jingjing Lian, Aiping Xu, Tao Chen, Yuan Chu, Yan Tang, Yanli Ni, Meidong Xu

**Affiliations:** 1 Endoscopy Center, Department of Gastroenterology, Shanghai East Hospital, Tongji University School of Medicine, Shanghai, China

A 48-year-old woman underwent a colonoscopy at our hospital for routine screening. During the procedure, a suspicious fistula was identified in the descending colon ( Fig. 1 ), with a brown metallic foreign body visible at the fistula opening as the endoscope approached ( Fig. 2 ). We grasped the exposed end of the foreign body with biopsy forceps and slowly pulled it into the intestinal lumen with great caution ( Video 1 ). Finally, the foreign body was successfully removed without any active bleeding and it was confirmed to be an intrauterine device (IUD) ( Fig. 3 ).

**Fig. 1:**
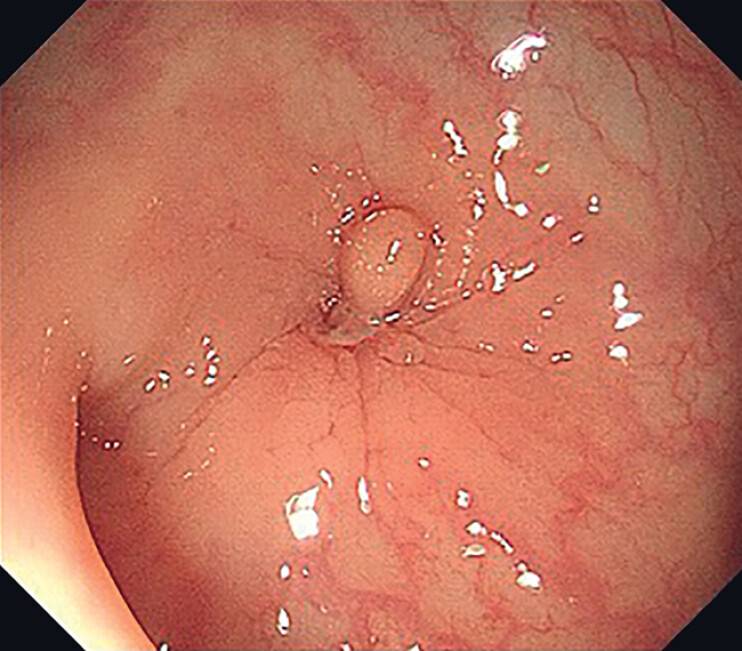
An unusual mucosal elevation was found in the descending colon.

**Fig. 2:**
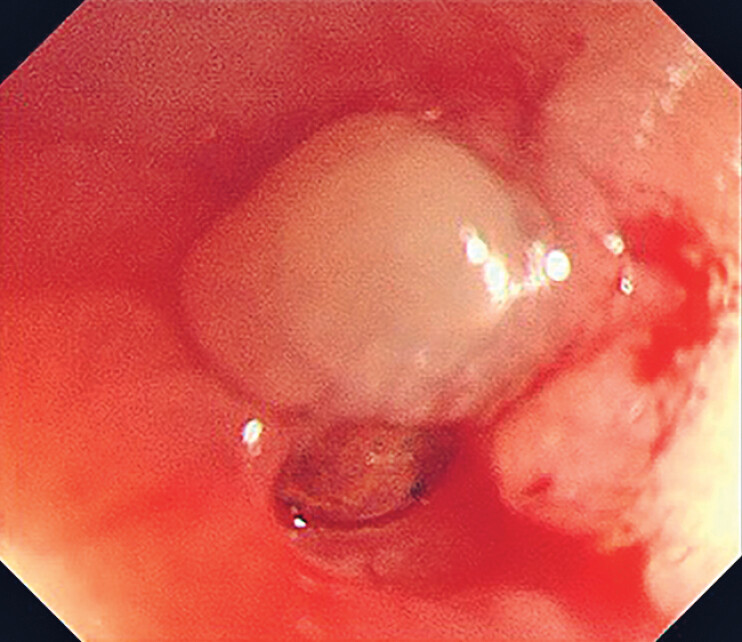
A brown metallic foreign body was visible at the suspicious fistula opening when the endoscope approached more closely.

The process of endoscopic removal of an intrauterine device transmigrating to the colonic wall.Video 1

**Fig. 3:**
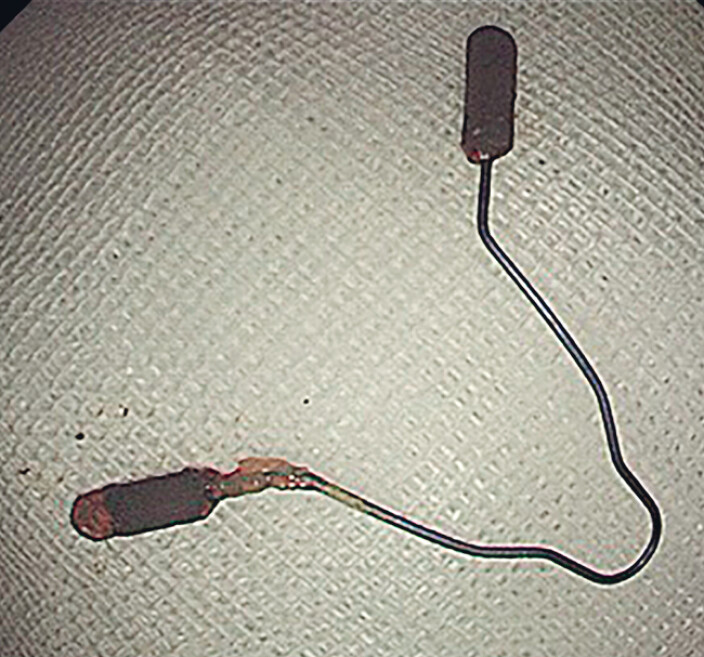
The foreign body was confirmed to be an intrauterine device after endoscopic removal.

Following the removal of the IUD, we reinserted the scope and confirmed that the fistula had closed, obviating the need for additional closure procedures. Further investigation into the patientʼs medical history revealed that she had undergone IUD placement at the age of 38. Apart from experiencing amenorrhea 3 months prior, she denied any other symptoms.

After the removal procedure, the patient did not report any abdominal pain or fever, and no additional treatment was deemed necessary. She was monitored in the hospital for 3 hours until an abdominal computed tomography scan confirmed the absence of perforation. During the 1-year follow-up period, no adverse events occurred.

The transmigration of an IUD to the colonic wall is an exceptionally rare complication, with the sigmoid colon being the most commonly observed migrating location. Surgical intervention is typically necessary in the majority of reported cases 1 2 . To the best of our knowledge, this is the first time the migrated IUD was successfully extracted solely using biopsy forceps without the need for additional measures. Endoscopic treatment is strongly recommended for patients with IUD transmigration to the colonic wall as long as there is no evidence of free perforation or associated complications.

Endoscopy_UCTN_Code_CCL_1AD_2AH

## Notes

Endoscopy E-Videos https://eref.thieme.de/e-videos
*E-Videos* is an open access online section of the journal *Endoscopy* , reporting on interesting cases and new techniques in gastroenterological endoscopy. All papers include a high-quality video and are published with a Creative Commons CC-BY license. Endoscopy E-Videos qualify for HINARI discounts and waivers and eligibility is automatically checked during the submission process. We grant 100% waivers to articles whose corresponding authors are based in Group A countries and 50% waivers to those who are based in Group B countries as classified by Research4Life (see: https://www.research4life.org/access/eligibility/ ). This section has its own submission website at https://mc.manuscriptcentral.com/e-videos .
